# Toolbox for the Extraction and Quantification of Ochratoxin A and Ochratoxin Alpha Applicable for Different Pig and Poultry Matrices

**DOI:** 10.3390/toxins14070432

**Published:** 2022-06-24

**Authors:** Barbara Streit, Tibor Czabany, Georg Weingart, Martina Marchetti-Deschmann, Shreenath Prasad

**Affiliations:** 1 BIOMIN Research Center, 3430 Tulln an der Donau, Austria; barbara.streit@dsm.com (B.S.); tibor.czabany@dsm.com (T.C.); georg.weingart@dsm.com (G.W.); 2 Institute of Chemical Technologies and Analytics, TU Wien, 1060 Vienna, Austria; martina.marchetti-deschmann@tuwien.ac.at

**Keywords:** mycotoxin, swine matrices, chicken matrices, liquid chromatography, tandem mass spectrometry, method validation

## Abstract

Ochratoxin A (OTA) is one of the major mycotoxins causing severe effects on the health of humans and animals. Ochratoxin alpha (OTα) is a metabolite of OTA, which is produced through microbial or enzymatic hydrolysis, and one of the preferred routes of OTA detoxification. The methods described here are applicable for the extraction and quantification of OTA and OTα in several pig and poultry matrices such as feed, feces/excreta, urine, plasma, dried blood spots, and tissue samples such as liver, kidney, muscle, skin, and fat. The samples are homogenized and extracted. Extraction is either based on a stepwise extraction using ethyl acetate/sodium hydrogencarbonate/ethyl acetate or an acetonitrile/water mixture. Quantitative analysis is based on reversed-phase liquid chromatography coupled to tandem mass spectrometry (LC-MS/MS). Method validation was successfully performed and the linearity, limit of quantification, accuracy, precision as well as the stability of the samples, were evaluated. The analyte recovery of the spiked samples was between 80 and 120% (80–150% for spiked concentrations ≤ 1 ng/g or ng/mL) and the relative standard deviation was ≤ 15%. Therefore, we provide a toolbox for the extraction and quantification of OTA and OTα in all relevant pig and poultry matrices.

## 1. Introduction

Mycotoxins are toxic secondary metabolites produced by filamentous fungi contaminating food and animal feed [1]. One of the major mycotoxins is ochratoxin A (OTA), which was first reported in *Aspergillus ochraceus* [2], but later also found in other *Aspergillus* and *Penicillum* species [3]. OTA is considered to be the most toxic member of the ochratoxin group consisting also of ochratoxin B and ochratoxin C [3]. There are various effects of OTA reported on the health of human and animals. It is a nephrotoxin and has also been shown to be hepatoxic, teratogenic, immunotoxic, and carcinogenic in various species [4,5,6,7,8,9,10]. In addition, it is classified by the International Agency of Research on Cancer (IARC) as a possible human carcinogen group 2B [11,12].

There is a broad range of exposure routes to OTA. Different types of food and feed can be contaminated, including cereals like wheat, rice, rye, maize, and barley [3]. Therefore, cereal-based products, raisins and wine, grapes, nuts, spices, legumes, but also coffee and beer are affected [3]. OTA has a high affinity to proteins such as serum albumin, which promotes accumulation in animal products [5]. The highest levels of OTA were found in porcine blood-based sausages or liver products, but also in other animal products such as milk or meat OTA can also be detected [3,5,13,14]. Considering the harmful effects of OTA on human and animal health and welfare, the European Commission has set maximum levels for OTA in several food products in the range of 0.5–80 µg/kg (EC, 1881/2006) [15], and recommendations for OTA in animal feed between 10–250 µg/kg (EC, 2016/1319) [16], to minimize the risk of exposure and assure product safety.

Ingested OTA can easily be absorbed and further distributed to different organs. Nevertheless, natural detoxification into less-/nontoxic metabolites, such as ochratoxin alpha (OTα) and phenylalanine, is possible by enzymes and microbes in the gut [17,18,19]. The concentration of OTA metabolites in biological samples is dependent on the OTA dose and exposure time, and generally lower compared to measured OTA concentrations in feed. Even if there are lower concentrations, estimating OTα along with OTA in biological samples would be useful to monitor OTA exposure. 

In human and animal matrices many methods for the detection of OTA are either based on liquid extraction from tissue or feed [20,21,22,23,24,25,26] or OTA levels are estimated by dried blood spot (DBS) analysis [27,28,29,30]. We provide here a comprehensive toolbox, which is easily established, for the extraction and reliable quantification of OTA and OTα in many different matrices. The methods are either based on the green solvent ethyl acetate or rather small volumes (<1 mL) of acetonitrile. Quantitative analysis is done by liquid chromatography coupled to tandem mass spectrometry (LC-MS/MS). The focus of this work is on animal matrices. Methods were developed and validated for the quantification of OTA and OTα in pig as well as chicken samples. The methods are applicable to many matrices of interest related to animals such as feed, feces/excreta, liver, kidney, muscle, skin, and fat. Furthermore, the extraction mechanism was adapted for liquid matrices such as urine or plasma samples. With the rising interest in DBS samples, this sampling method was included as well. Validation parameters cover the accuracy and precision of analysis as well as the linear quantification ranges, limits of quantification (LOQ), and limits of detection (LOD) for each matrix. The stability of extracted samples ready for measurement was evaluated at different storage temperatures and durations for each matrix. The presented methods are suitable for routine measurements to monitor OTA exposure of animals and the effects of OTA-mitigating agents supplemented in feed. 

## 2. Results

**Figure 1:**
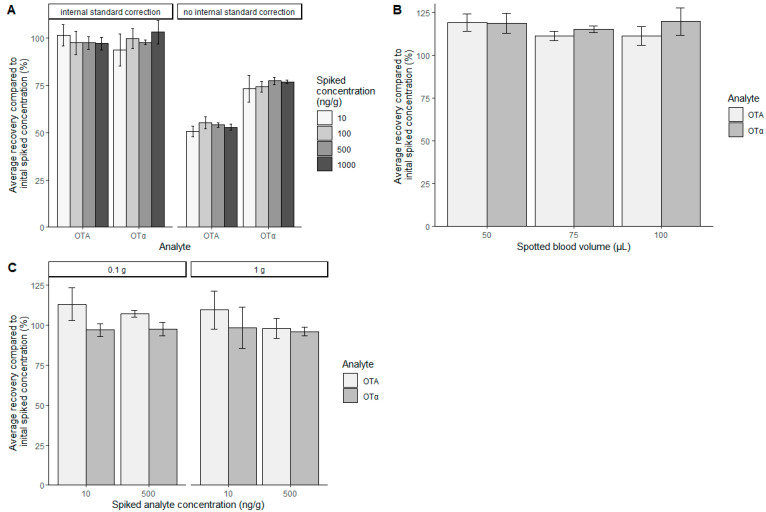
(**A**) Comparison between analyte recovery of OTA and OTα in pig feed with and without internal standard correction using ^13^C-labelled analytes. (**B**) Influence of the blood volume spotted on protein saver cards on the analyte recovery for OTA and OTα. (**C**) Comparison of analyte recovery of different sample amounts for chicken excreta. For extraction, 1 g or 0.1 g excreta were used.

The aim of this work was to develop simple methods for the extraction and quantification of OTA and OTα in different pig and chicken matrices. The matrices of interest were feces or excreta, urine (only available from pig), plasma, DBS, skin and fat (only from chicken), liver, kidney, muscle, and feed for pigs and poultry. For the method development and validation, blank matrices were homogenized and spiked with OTA and OTα at different levels, covering the full linear range of quantification for each different matrix. Afterwards, the samples were extracted using ethyl acetate followed by re-extraction in a reduced amount of sodium hydrogencarbonate solution, and another re-extraction in a reduced amount of ethyl acetate. A similar stepwise liquid/liquid extraction was already described by Monaci et al. [21] or Giacomo et al. [20]. Our simplified procedure allows the concentration of the analytes and clean-up of the samples to minimize matrix effects during this stepwise extraction. For DBS, a direct extraction using acetonitrile (ACN)/water (70/30 *v/v*) mixture worked best. Regardless of the matrix extracted, the analyte concentration was determined by reversed phase LC-MS/MS using external calibration in neat solvent. Possible extraction losses and matrix effects during ionization were compensated using ^13^C-labelled OTA and OTα as internal standards, which were added to the samples prior to extraction. Figure 1A shows exemplarily the analyte recoveries with and without internal standard correction for pig feed. Without internal standard correction, the analyte recovery is approximately 55% and 75% for OTA and OTα, respectively. Applying internal standard correction for each analyte increases the analyte recovery to 80–120% of the initially spiked concentration. As the blood volume dried on protein saver cards can influence the results, blood was spiked with analyte, and different blood volumes (50 µL, 75 µL, and 100 µL) were spotted and extracted (Figure 1B). No influence of the spotted blood volume on the analyte recovery was visible. Especially in the case of chicken excreta, the available sample quantity can be very limited. Therefore, the amount of sample to be extracted can be reduced to 100 mg in combination with scale-down of all following steps. The same analyte recoveries for OTA and OTα are achieved for both sample amounts, at 1 g and 0.1 g, respectively (Figure 1C).

To determine if the method is fit for its intended purpose, a single-laboratory method validation was carried out, and the linearity of the calibration function, intraday and interday precision, accuracy, LOQ, LOD, specificity, and analyte stability in processed samples were evaluated. 

**Figure 2:**
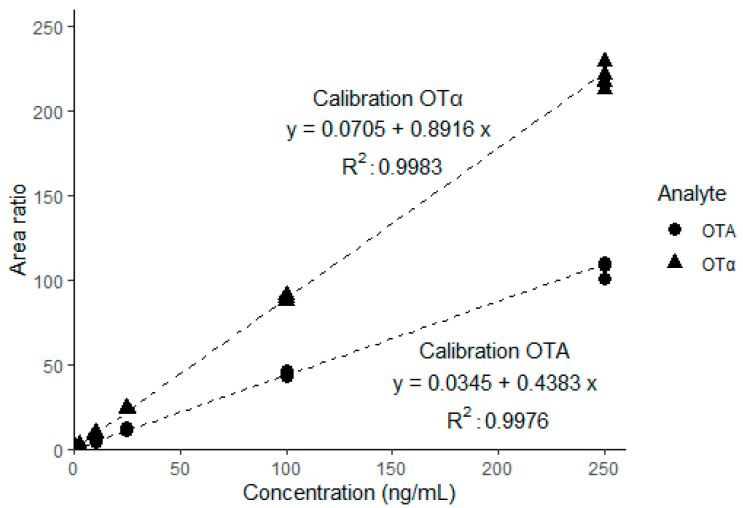
Calibration of OTA and OTα using four independent dilutions (seven individual calibration points) in neat solvent from 0.25 ng/mL to 250 ng/mL. ^13^C-labelled analytes were used as internal standard for correction.

The linearity of the external calibration for OTA and OTα was assessed by preparing four individual calibration series from seven individual concentration levels between 0.25 ng/mL and 250 ng/mL in neat solvent (Figure 2). For the calibration, internal standard correction (^13^C-labelled OTA and OTAα) was applied for the linear regression with a weighting factor of 1/analyte concentration, as recommended by Gu et al. [31]. The calculated squared correlation coefficient (R^2^) was > 0.99.

Accuracy and precision were determined in all matrices using blank samples spiked with OTA and OTα. Samples with different spiking levels were extracted on two independent days to calculate the interday precision. The intraday precision was calculated from an independent triplicate prepared on the same day. The intraday precision (calculated from a triplicate on the same day) and interday precision (calculated from independent extractions on two days, as it will be done in following routine measurements) showed a relative standard deviation (RSD) ≤ 15% for all matrices. For all different matrices evaluated, the accuracy of the method was between 81% and 147%. Analyte recovery of over 120% was determined only for low-level concentrations at the LOQ for poultry plasma samples, and liver, kidney, and muscle samples from pigs. Therefore, the set acceptance criteria for the analyte recovery of 80–120% (80–150% for spiked concentrations ≤ 1 ng/g or ng/mL) of the spiked concentration were met. To fulfill all validation criteria according to accuracy and precision, ^13^C-labelled internal standard correction for OTA and OTα was used for all matrices except for DBS of pigs. DBS of pigs showed already without internal standard correction analyte recoveries between 88–116%, and therefore internal standard correction was not necessary. Table 1 and Table 2 summarize all evaluated validation parameters for pig and poultry matrices. 

The LOQ was estimated by spiking experiments. It is the smallest measured content of the analyte above which the determination can be made with a specified accuracy and precision, as recommended by the Guidance for Industry 208 from the Food and Drug Administration [32]. The LOD was estimated by calculating the analyte concentrations, which delivered an S/N ratio of 3/1 based on the S/N ratio of the analytes at the LOQ.

The specificity of the method is given by the retention time and the multiple reaction monitoring (MRM) transitions. Blank samples were extracted and measured to evaluate the endogenous response. No interfering peaks were detected at the specific retention time of OTA at 1.6 min and OTα at 1.3 min, except for pig plasma and pig DBS samples. In these samples, a small peak at the specific retention time of OTA was found, with an approximate S/N of LOD. As this peak was visible in all measured MRM transitions for OTA, we assume that this is not an interfering peak, but rather the analyte itself. We considered that the used plasma and blood samples were not completely OTA-free.

As analyte stability in processed samples is crucial for reliable results, the analyte stability of OTA and OTα in ready-to-inject samples was evaluated at different temperatures and storage periods. The analytes were stable in all evaluated matrices. At room temperature after 7 days of storage, slightly higher recoveries (up to 125% compared to the initial measured concentration) were observed in poultry matrices. All other tested storage conditions fulfilled the acceptance criteria of an analyte recovery of 80–120% compared to the initial measured value. Nevertheless, for long-term storage of processed samples, storage at −20 °C would be recommended.

## 3. Discussion

The intention of this work was to develop and validate methods for extraction and quantification of OTA and OTα in different pig and poultry matrices. The European Commission (EC/2016/1319) has published recommendations for OTA in feed, with maximum thresholds of 50 µg/kg for pig feed and 100 µg/kg for poultry feed [16]. Considering these contamination levels of OTA in the feed, we expected OTA levels for example in plasma to be in the low ng/mL range [33,34]. We present methods applicable for pig and poultry with similar extraction procedures for matrices such as feed, feces/excreta, urine, plasma, DBS, liver, kidney, muscle, skin, and fat. The primary goal of the method development was the quantitative determination of OTA and OTα at biologically relevant concentration levels. 

There are already many methods described for the detection and quantification of OTA, and sometimes also OTα, in different matrices. The methods presented here are similar to those described by Monaci et al. [21] or Giacomo et al. [20], as the same step-wise extraction and clean-up procedure using solid/liquid and liquid/liquid extraction mechanisms were used. However, in contrast to the already described protocols, our method is applicable to a larger variety of matrices and also simpler, as some extraction steps have been modified. Monaci et al. as well as Giacomo et al. also use the three-phase solid/liquid/liquid extraction system, but the tissue samples are extracted twice. So removal of the organic phase is required. We simplified the extraction by only applying a single extraction of the matrix. Then sample preparation is continued with a defined aliquot of the organic phase, which can be easily pipetted. Furthermore, other methods rely on the reduction of the sample volume to a specific amount [20]. In our methods, clean-up and concentration of analytes are achieved by re-extraction in reduced volumes of sodium hydrogencarbonate solution and ethyl acetate. Moreover, these steps also provide fat-removal from the samples. Therefore, the use of expensive SPE columns for sample clean-up, as reported for liver samples by Monaci et al. [21], is not necessary. Other methods for the extraction of OTA and OTα are often based on halogenated solvents such as dichloromethane [35] or an enzymatic digestion [36]. The methods described here used the green solvent ethyl acetate for the main extraction of the analytes from the matrix. For the extraction of DBS using ethyl acetate was not successful. Instead of using this green solvent, rather small volumes (<1 mL) of ACN/water mixtures were used for the extraction of DBS and showed good results. 

As sometimes the available quantity of samples is very limited, the robustness of the method regarding the sample amount was evaluated. Starting from 1 g or 0.1 g of excreta, the extraction parameters were scaled down for the smaller sample size. However, no influence on the results was observed, confirming the ruggedness of our method. Moreover, different blood volumes dried on protein saver cards were evaluated regarding analyte recovery. The same results were obtained, regardless of the spotted blood volume. This is in accordance with results of Osteresch et al., where different blood volumes had almost no impact on the results for OTA [29]. The same principle of extraction is applied to solid samples such as tissue or feces as well as liquid samples such as urine and plasma. Hence, we provide here a widely applicable, robust toolbox for the extraction and quantification of OTA and OTα in all relevant pig and poultry matrices.

## 4. Conclusions and Outlook

The detection of OTA and its metabolite OTα in important pig and poultry matrices was established and validated. The developed methods allow the extraction and quantification of OTA and OTα in feed, plasma, DBS, liver, kidney, and muscle samples from pigs and chickens. Furthermore, extraction protocols for feces and urine samples from pigs as well as excreta, skin, and fat samples from chickens are available. Extraction is similar for all matrices, and either based on the green solvent ethyl acetate or on rather small volumes of ACN/water. Quantitative analysis of the analytes is done using LC-MS/MS. Method development was focused on the detection of biologically relevant concentrations. Therefore, the LOQ is between 0.5 ng/g in tissue matrices and 10 ng/g in feed or feces/excreta. The accuracy and precision of the methods were evaluated, and the analyte recovery was between 80 and 120% (80–150% for spiked concentrations ≤ 1 ng/g or ng/mL) of the initially spiked concentration and the RSD was ≤ 15%. Analyte stability in processed samples was tested at room temperature, at 4 °C and −20 °C for different storage periods. For long term storage −20 °C is recommended. The presented methods are easily established and feasible to monitor OTA exposure or for the use in feeding trials of pig and poultry to evaluate OTA-mitigating feed additives.

## 5. Materials and Methods

### 5.1. Chemicals and Reagents

The analytical standards for OTA, OTα, and ^13^C-labelled OTA as internal standard were obtained from Romer Labs (Tulln an der Donau, Austria). The ^13^C-labelled OTα was produced in-house by enzymatic degradation of ^13^C-labelled OTA into ^13^C-labelled OTα and phenylalanine. An analytical standard for creatinine was purchased from Merck (Darmstadt, Germany). All standards were stored as recommended by the supplier. Ortho-phosphoric acid 85%, formic acid, ACN HPLC grade, and acetic acid were obtained from VWR (Vienna, Austria). Ethyl acetate HPLC grade and ACN HPLC-MS/MS grade were purchased from Chem-Lab (Zedelgem, Belgium). Methanol (MeOH) LC-MS grade was bought from Honeywell (Charlotte, NC, USA). Sodium hydrogencarbonate and Whatman 903 protein saver cards were obtained from Merck (Darmstadt, Germany). Ultrapure water was produced in-house using a Milli-Q IQ 7000 water purification system from Merck (Darmstadt, Germany).

### 5.2. Preparation of Spiking Solutions and Spiking of the Samples

Standard stock solutions for OTA, OTα, and ^13^C-labelled OTA were purchased with a concentration of 10 µg/mL already dissolved in ACN. The ^13^C-labelled OTα was prepared by proprietary enzymatic degradation of ^13^C-labelled OTA. Progress of conversion of ^13^C-OTA to ^13^C-OTα was checked by LC-MS/MS. After complete conversion ^13^C-OTα was extracted five times with ethyl acetate. The combined ethyl acetate phases were dried completely under reduced pressure and reconstituted in ACN to a final concentration of 6.34 µg/mL. The same enzymatic reaction can be performed with any other OTA hydrolyzing enzymes, such as Carboxypeptidase A, under suitable reaction conditions.

Stock solutions of OTA and OTα were mixed and used directly or optionally further diluted in ACN/water/formic acid (50/49/1 *v/v/v*) to the respective target concentrations between 1 µg/mL and 10 ng/mL. The prepared solutions were used for spiking the matrices at different concentration levels. Internal standard was prepared by dilution and mixing of ^13^C-OTA and ^13^C-OTα in ACN/water/formic acid (50/49/1 *v/v/v*) to 1 µg/mL, 0.25 µg/mL, or 0.1 µg/mL. Samples were spiked with internal standard during sample preparation, as described below. Further dilutions of internal standard were prepared and used for the external calibration in neat solvent.

### 5.3. Biological Samples

For method development and validation, urine, plasma, blood, feces, excreta, feed (based on wheat–barley–corn–soy diet for pig and corn–soy diets for poultry), liver, kidney, muscle, skin, and fat samples were obtained in-house from the center of applied animal nutrition (BIOMIN, Tulln an der Donau, Austria) or from the local slaughterhouse. Blood was analyzed using dried blood spots. All samples except feed, lyophilized feces or excreta, and DBS samples were stored at −20 °C until sample preparation. Feed, lyophilized feces or excreta samples were stored at room temperature in the dark until sample preparation. DBS were stored at 4 °C in the dark after drying overnight at room temperature. For all matrices quantitative determination was based on external calibration in neat solvent.

#### 5.3.1. Urine (Pig) and Plasma Samples (Pig and Poultry)

The concentration of analytes in urine depends, among others, on the amount of water the animal was drinking. To reduce variation in the results, urine samples were diluted to a specified concentration of creatinine. Determination of the creatinine concentration of each urine sample, and afterwards the individual dilution factor, urine samples were brought to room temperature, mixed shortly on a vortex shaker, and diluted 1:10,000 in ultrapure water. Creatinine concentration was determined by LC-MS/MS in positive mode. An Agilent 1290 Infinity II system was coupled to a Sciex Triple Quad 5500 mass spectrometer. Chromatographic separation was achieved on a Gemini 5 µm C18 column 150 × 4.6 mm with a suitable precolumn from Phenomenex (Aschaffenburg, Germany). The column compartment was heated to 30 °C and the injection volume was set to 2 µL. Solvent A consisted of MeOH/water/acetic acid (40/59.8/0.2 *v/v/v*) and MeOH/acetic acid (99.8/0.2 *v/v*) was used as solvent B. Flow rate was set to 0.8 mL/min and a gradient was used during the chromatographic run, containing 0 min–1.6 min 0% B constant, 1.6 min–1.65 min linear gradient to 100% B, 1.65 min–2 min 100% B constant, and 2 min–2.05 min linear gradient to 0% B. The total run time was 3 min. The measured MRM transitions were 114 > 86 (collision energy 15 volts, declustering potential 20 volts) as quantifier and 114 > 44 (collision energy 15 volts, declustering potential 20 volts) as qualifier. The retention time of creatinine was 1.36 min. Quantitative determination of creatinine was based on a serial dilution of standards in MeOH/water (10/90 *v/v*). After determination of the creatinine values, urine samples were diluted individually with ultrapure water to 10 µM creatinine. If the creatinine value was below 10 µM, the samples were used directly. 

The samples (diluted urine samples to 10 µM creatinine or plasma samples) were thawed and mixed shortly on a vortex shaker. An aliquot of 200 µL was transferred to a fresh Eppendorf tube and spiked with 8 µL of internal standard solution containing 1 µg/mL ^13^C-OTA and ^13^C-OTα. Then 800 µL extraction solution consisting of ethyl acetate/phosphoric acid 85% (99/1 *v/v*) were added and mixed by manual shaking to make sure that the samples did not stick to the bottom of the vial, before mixing vigorously for 10 min on a vortex shaker with adapter for 2 mL reaction tubes. The samples were centrifuged for 5 min at 19,000 rcf. Finally, 50 µL of the supernatant were transferred to an HPLC vial with 0.2 mL silanized insert and mixed with 50 µL ACN. 

#### 5.3.2. Feed, Feces, and Excreta

Fresh feces and excreta were freeze dried. For extraction, 1 g of the sample was weighed in a 50 mL falcon tube and spiked with 30 µL of the mixed internal standard solution containing 1 µg/mL ^13^C-OTA and ^13^C-OTα. Then 6 mL 1 M phosphoric acid were added and mixed shortly, followed by the addition of 30 mL ethyl acetate. The samples were extracted on an end-over-end shaker for 60 min at 80 rpm. The samples were centrifuged for 10 min at 3200 rcf. Afterwards, 8 mL of the supernatant were transferred to a fresh 50 mL falcon tube and mixed with 4 mL 0.1 M sodium hydrogencarbonate solution (pH 8.2). The samples were mixed for 1 min on the end-over-end shaker at 80 rpm and centrifuged for 10 min at 3200 rcf. Then 3 mL of the water phase were transferred to a fresh 15 mL falcon tube and mixed with 70 µL ortho-phosphoric acid 85%. Finally, 1.5 mL ethyl acetate were added to the sample and the samples were mixed for 1 min on the end-over-end shaker at 80 rpm. The samples were centrifuged for 10 min at 3200 rcf and 50 µL of the supernatant were transferred to an HPLC vial with a 0.2 mL silanized insert and mixed with 50 µL ACN.

To test the robustness of the method, the used sample amount for extraction was reduced to 100 mg and all following steps were adapted to this smaller amount. 

#### 5.3.3. Liver, Kidney, Muscle, Skin, and Fat Samples

Fresh tissue samples were frozen and stored at −20 °C in sealed bags. For homogenization, the samples were cooled in liquid nitrogen for approximately 1 min and pulverized using a ball mill (MM 400 from Retsch, Haan, Germany) for 30 s with 30 Hz in two cycles. For extraction, 1 g of the sample was weighed in a 50 mL falcon tube and spiked with 30 µL of the mixed internal standard solution containing 0.1 µg/mL ^13^C-OTA and ^13^C-OTα. The same procedure as already described in 5.3.2 for feed, feces, and excreta samples was used for further sample preparation. As the expected analyte concentrations are lower in tissue compared to feed or feces/excreta, an additional step for analyte concentration was added. Therefore, 1 mL of the supernatant after the second ethyl acetate extraction step was transferred to a fresh Eppendorf tube and dried completely under reduced pressure. The samples were reconstituted in 100 µL ACN/water/formic acid (50/49/1 *v/v/v*) in the ultrasonic bath for 10 min. Afterwards the samples were mixed vigorously for 10 min on a vortex shaker. Subsequently, the samples were centrifuged for 5 min at 19,000 rcf before 50 µL of the supernatant were transferred to an HPLC vial with a 0.2 mL silanized insert. 

#### 5.3.4. Dried Blood Spots

For the preparation of DBS, 50 µL whole blood were spotted on the protein saver cards and dried overnight at room temperature. To test the influence of the spotted volume, in addition 75 µL and 100 µL blood were spotted.

For DBS of pigs, the whole blood spot (50 µL, 75 µL or 100 µL) was cut out from the protein saver card and placed in a 1.5 mL Eppendorf tube. Then 800 µL extraction solution containing ACN/water (70/30 *v/v*) were added and mixed. The samples were placed on an end-over-end shaker for 60 min at 80 rpm. Afterwards, 100 µL of the liquid were transferred to an HPLC vial with a 0.2 mL silanized insert. 

A similar approach was used for DBS from chicken. Here, 50 µL of whole blood were spotted on protein saver cards and dried overnight. The entire spot was cut out and placed in a 1.5 mL Eppendorf tube. Additionally, 10 µL mixed internal standard solution containing 250 ng/mL of ^13^C-OTA and ^13^C-OTα were spiked in the tubes. Then 800 µL extraction solution of ACN/water (70/30 *v/v*) were added and mixed. The samples were placed for 60 min on an end-over-end shaker at 80 rpm. An aliquot of 500 µL of the supernatant was dried completely under reduced pressure at 60 °C and reconstituted afterwards in 62.5 µL ACN/water/formic acid (50/49/1 *v/v/v*). Samples were mixed vigorously for 10 min on a vortex shaker and afterwards centrifuged for 10 min at 19,000 rcf. Finally, 50 µL of the supernatant were transferred to an HPLC vial with a 0.2 mL silanized insert. 

### 5.4. Chromatography and LC-MS/MS Parameters

Quantification was based on reversed-phase LC-MS/MS in negative MRM mode. Therefore, an Agilent 1290 Infinity II system was coupled to a Sciex QTRAP 6500+ mass spectrometer. Chromatographic separation was achieved on a Kinetex 2.6 µm EVO C18 column 150 × 2.1 mm with a suitable precolumn from Phenomenex (Aschaffenburg, Germany). The temperature of the column oven was set to 40 °C and 4 µL injection volume were used. The optimum chromatographic conditions were achieved with eluent A water/ACN (95/5 *v/v*) containing 0.1% formic acid and eluent B ACN/water (95/5 *v/v*) containing 0.1% formic acid. A gradient was used for separation: 0 min–0.25 min 20% B constant, 0.25 min–2 min linear gradient to 100% B, 2 min–2.50 min 100% B constant, and 2.50 min–2.51 min linear gradient to 20% B. The total run time was 3 min. Over the whole gradient the flow rate was set to 1 mL/min. The measured MRM transitions were optimized using direct infusion of the analytes. The optimized parameters for each analyte are listed in Table 3.

### 5.5. Method Validation 

For method validation, blank matrix was spiked at two levels, extracted, and measured. The following parameters were evaluated: linearity, intraday and interday precision, accuracy, LOQ, LOD, specificity, and analyte stability in processed samples.

#### 5.5.1. Linearity

Linearity was assessed by preparing four individual calibration series through diluting defined concentrations of OTA, OTα, ^13^C-OTA, and ^13^C-OTα in ACN/water/formic acid (50/49/1 *v/v/v*) in the range from 0.25 ng/mL to 250 ng/mL. Linear regression was performed with a weighting factor of 1/analyte concentration, as recommended by Gu et al. [31]. The R^2^ was calculated and the acceptance criteria was set at R^2^ >0.99.

#### 5.5.2. Accuracy and Precision

Accuracy and precision were evaluated by analyzing blank samples, which were spiked with defined concentrations of OTA and OTα at two days at two different levels. The accuracy was calculated using the following equation:

(1) $$\mathrm{Accuracy}\;\left(\%\right)=\frac{\mathrm{measured}\;\mathrm{concentration}\;\left(\frac{ng}{g}or\frac{ng}{mL}\right)}{\mathrm{spiked}\;\mathrm{concentration}\;\left(\frac{ng}{g}\;or\frac{ng}{mL}\right)}\times 100$$

The acceptance criteria for accuracy were for spiking levels above 1 ng/g or ng/mL an analyte recovery of 80–120% of the initial spiked concentration. For spiking levels less or equal to 1 ng/g or ng/mL 80–150% analyte recovery was accepted. For intraday precision, the samples were spiked and analyzed in triplicate on the same day. For the interday precision two independent extractions were analyzed, as planned for further routine analysis. For the intraday and interday precision the acceptance criteria were set to RSD ≤ 15%. To calculate the RSD, the following equation was used:

(2) $$RSD\;\left(\%\right)=\frac{\sqrt{\frac{\sum {\left(\mathrm{measured}\;\mathrm{concentration}\;\left(\frac{ng}{g}\;or\frac{ng}{mL}\right)-\mathrm{average}\;\mathrm{concentration}\;\left(\frac{ng}{g}\;or\frac{ng}{mL}\right)\;\right)}^{2}}{\mathrm{number}\;of\;\mathrm{replicates}-1}}}{\mathrm{spiked}\;\mathrm{concentration}\;\left(\frac{ng}{g}\;or\frac{ng}{mL}\right)}\times 100\;$$

#### 5.5.3. Limit of Quantification and Limit of Detection

For the determination of the LOQ, spiking experiments were used. As defined in the Guidance for Industry 208, LOQ is the smallest measured content of analyte which can be determined with specified accuracy and precision [32]. LOD was estimated by calculating the analyte concentrations which delivered a S/N ratio of 3/1 based on the S/N ratio of the analytes at LOQ.

#### 5.5.4. Selectivity

Selectivity is given by the retention time and the specific MRM transitions of the analytes. Furthermore, the endogenous response of blank samples was evaluated. A signal of interferences at the retention time of the analyte below a S/N ≤ 3:1 was acceptable.

#### 5.5.5. Analyte Stability in Processed Samples

Samples were extracted and extracts ready to inject were stored at different storage conditions in triplicate. The stability in process samples was evaluated at room temperature up to 7 days, in the fridge at 4 °C up to 7 days, and in the freezer at −20 °C up to 7 days for liver, kidney, and muscle samples, as low analyte concentrations are expected in combination with a high matrix content after drying and reconstitution, or at −20 °C up to 30 days for all other matrices. The acceptance criteria were 80–120% analyte recovery compared to the initial measured concentration.

## Figures and Tables

**Table 1:** Summary of validation parameters for pig matrices.

		Feces		Urine		Plasma		DBS		Liver		Kidney		Muscle		Feed	
Method Parameters		OTA	OTα	OTA	OTα	OTA	OTα	OTA	OTα	OTA	OTα	OTA	OTα	OTA	OTα	OTA	OTα
Range of analysis (ng/g or ng/mL)		10–500	10–500	5.0–500	5.0–500	5.0–500	5.0–500	5.0–500	5.0–500	0.50–100	0.50–100	0.50–100	0.50–100	0.50–100	0.50–100	10–1000	10–1000
LOQ (ng/g or ng/mL)		10	10	5.0	5.0	5.0	5.0	5.0	5.0	0.50	0.50	0.50	0.50	0.50	0.50	10	10
LOD (ng/g or ng/mL)		3.0	3.0	1.5	1.5	1.5	1.5	1.5	1.5	0.15	0.15	0.15	0.15	0.15	0.15	3.0	3.0
^13^C internal standard correction		yes	yes	yes	yes	yes	yes	no	no	yes	yes	yes	yes	yes	yes	yes	yes
Analyte recovery (%)	Low level ^1^	106–113	110–120	88–91	83–97	98–120	90–103	88–106	93–110	101–130	90–104	108–115	130–147	95–114	123–142	96–107	85–101
	High level ^2^	90–98	108–117	93–102	96–97	94–104	93–114	97–114	110–116	89–93	87–91	95–106	93–97	84–113	88–100	94–101	92–110
Max. intraday precision (*n* = 3) RSD (%)		3.7	5.1	4.1	7.0	6.8	7.7	4.5	7.6	13	6.0	6.5	9.6	14	8.4	5.8	8.4
Max. interday precision (*n* = 2) RSD (%)		5.3	7.4	12	2.6	7.2	11	12	8.7	11	2.3	1.3	6.3	13	6.6	2.4	4.8
Processed sample stability (%)	RT (2 days)	100–101	95–100	94–100	102–103	90–97	93–95	116–120	112–116	93–98	106–110	94–99	104–106	94–101	99–108	93–98	98–109
	4 °C (7 days)	102–112	104–112	97–120	81–89	90–99	90–96	97–106	94–102	104–114	99–103	100–102	108–110	97–114	101–108	99–113	110–118
	−20 °C (30 days) ^3^	95–98	100–108	85–93	104–106	96–100	96–98	109–110	109–113	103–108	103–114	100–117	96–105	95–107	100–104	89–99	106–114

^1^ spiking at the stated lower range of analysis, which is equal to the LOQ for this matrix; ^2^ spiking at the stated upper range of analysis; ^3^ in liver, kidney, and muscle samples, the stability was tested for 7 days

**Table 2:** Summary of validation parameters for poultry matrices.

		Excreta		Plasma		DBS		Liver		Kidney		Muscle		Skin and Fat		Feed	
Method Parameters		OTA	OTα	OTA	OTα	OTA	OTα	OTA	OTα	OTA	OTα	OTA	OTα	OTA	OTα	OTA	OTα
Range of analysis (ng/g or ng/mL)		10–1000	10–1000	1.0–1000	1.0–1000	1.0–500	1.0–500	0.50–200	0.50–200	0.50–200	0.50–200	0.50–200	0.50–200	0.50–200	0.50–200	10–1000	10–1000
LOQ (ng/g or ng/mL)		10	10	1.0	1.0	1.0	1.0	0.5	0.5	0.5	0.5	0.5	0.5	0.5	0.5	10	10
LOD (ng/g or ng/mL)		3.0	3.0	0.30	0.30	0.30	0.30	0.15	0.15	0.15	0.15	0.15	0.15	0.15	0.15	3.0	3.0
^13^C internal standard correction		yes	yes	yes	yes	yes	yes	yes	yes	yes	yes	yes	yes	yes	yes	yes	yes
Accuracy range (%)	Low level ^1^	96–116	90–113	102–122	81–99	97–103	91–104	89–95	89–116	89–101	85–115	81–101	89–114	99–114	88–98	92–107	94–100
	High level ^2^	93–105	93–98	103–110	102–108	87–97	88–101	96–105	94–102	91–105	98–104	94–104	94–105	85–99	85–90	98–107	94–105
Max. intraday precision (*n* = 3) RSD (%)		12	13	10	9.8	4.4	5.4	5.1	3.2	6.8	13	10	13	6.2	2.6	5.0	3.3
Max. interday precision (*n* = 2) RSD (%)		5.4	6.9	1.7	3.7	7.5	5.5	3.0	15	4.5	2.1	2.0	7.5	10	6.7	10	6.0
processed sample stability (%)	RT (7 days)	101–104	102–106	89–99	10–114	94–99	97–101	98–121	82–100	102–109	85–97	99–125	93–99	93–98	96–98	94–107	97–101
	4 °C (7 days)	101–105	90–106	86–100	100–103	103–106	103	98–99	88–97	105–109	90–98	103–111	92–106	92–94	96–99	98–99	100–103
	−20 °C (30 days)	93–97	98–101	85–95	95–117	99–101	99–101	97–108	97–100	99–103	100–103	95–103	97–100	93	98–106	99–105	99–101

^1^ spiking at the stated lower range of analysis, which is equal to the LOQ for this matrix; ^2^ spiking at the stated upper range of analysis.

**Table 3:** Measured MRM transitions for each analyte.

Analyte	Measured Form	Precursor Ion (*m/z*)	Quantifier Ion ^1^ Qualifier Ions (*m/z*)	Declustering Potential (V)	Collision Energy (V)	Collision Cell Exit Potential (V)	Entrance Potential (V)	Retention Time (min)
OTA	[M−H]^−^	402	167 358 211	−85	−46 −26 −36	−17 −21 −23	−10	1.60
OTα	[M−H]^−^	255	167 211 123	−25	−34 −22 −40	−19 −13 −13	−10	1.30
^13^C-OTA	[M−H]^−^	422	175 377 221	−85	−48 −28 −38	−11 −23 −13	−10	1.60
^13^C-OTα	[M−H]^−^	266	175 221 130	−55	−32 −22 −40	−19 −13 −13	−10	1.30

^1^ underline indicates the ion used quantification, all other ions are used as qualifier.

## Data Availability

Not applicable.
